# Impact of COVID-19 on Child Maltreatment: Income Instability and Parenting Issues

**DOI:** 10.3390/ijerph18041501

**Published:** 2021-02-05

**Authors:** Janet Yuen-Ha Wong, Abraham Ka-Chung Wai, Man Ping Wang, Jung Jae Lee, Matthew Li, Jojo Yan-Yan Kwok, Carlos King-Ho Wong, Anna Wai-Man Choi

**Affiliations:** 1 School of Nursing, The University of Hong Kong, Hong Kong 999077, China; janetyh@hku.hk (J.Y.-H.W.); mpwang@hku.hk (M.P.W.); leejay@hku.hk (J.J.L.); mwli0503@connect.hku.hk (M.L.); jojoyyk@hku.hk (J.Y.-Y.K.); 2 Emergency Medicine Unit, LKS Faculty of Medicine, The University of Hong Kong, Hong Kong 999077, China; awai@hku.hk; 3 Department of Pharmacology and Pharmacy and Department of Family Medicine and Primary Care, LKS Faculty of Medicine, The University of Hong Kong, Hong Kong 999077, China; carlosho@hku.hk; 4 Department of Social and Behavioral Sciences, City University of Hong Kong, Hong Kong 999077, China

**Keywords:** COVID-19, child maltreatment, income instability, job loss, parenting

## Abstract

*Introduction*: Children are widely recognized as a vulnerable population during disasters and emergencies. The COVID-19 pandemic, like a natural disaster, brought uncertainties and instability to the economic development of the society and social distancing, which might lead to child maltreatment. This study aims to investigate whether job loss, income reduction and parenting affect child maltreatment. *Methods***:** We conducted a cross-sectional online survey of 600 randomly sampled parents aged 18 years or older who had and lived with a child under 10 years old in Hong Kong between 29 May to 16 June 2020. Participants were recruited from a random list of mobile phone numbers of a panel of parents. Of 779 recruited target parents, 600 parents completed the survey successfully via a web-based system after obtaining their online consent for participating in the survey. *Results*: Income reduction was found significantly associated with severe (OR = 3.29, 95% CI = 1.06, 10.25) and very severe physical assaults (OR = 7.69, 95% CI = 2.24, 26.41) towards children. Job loss or large income reduction were also significantly associated with severe (OR= 3.68, 95% CI = 1.33, 10.19) and very severe physical assaults (OR = 4.05, 95% CI = 1.17, 14.08) towards children. However, income reduction (OR = 0.29, 95% CI = 0.15, 0.53) and job loss (OR = 0.47, 95% CI = 0.28, 0.76) were significantly associated with less psychological aggression. Exposure to intimate partner violence between parents is a very strong and significant factor associated with all types of child maltreatment. Having higher levels of difficulty in discussing COVID-19 with children was significantly associated with more corporal punishment (OR = 1.19, 95% CI = 1.05, 1.34), whereas having higher level of confidence in managing preventive COVID-19 behaviors with children was negatively associated with corporal punishment (OR = 0.87, 95% CI = 0.76, 0.99) and very severe physical assaults (OR = 0.74, 95% CI = 0.58, 0.93). *Conclusions*: Income instability such as income reduction and job loss amplified the risk of severe and very severe child physical assaults but protected children from psychological aggression. Also, confidence in teaching COVID-19 and managing preventive COVID-19 behaviors with children was significantly negatively associated with corporal punishment during pandemic.

## 1. Introduction

The coronavirus disease 2019 (COVID-19) pandemic has changed all walks of life in 2020, among which economic uncertainties and instability have become a global concern. While global economic recovery is yet to come, child maltreatment came to public concern. Child maltreatment leads to serious health consequences, including mental health problems [1], morbidity of chronic illness and early mortality [2]. COVID-19 is considered as a natural crisis, which has disrupted the economic activities and society operation, leading to adverse economic conditions and job losses. Historically, in crisis and economic downturns, child physical and psychological abuse surged. The Great Recession would be an example [3]. However, at the time of writing, there has been no report on the impact of the COVID-19 pandemic on child maltreatment related to the income instability or unemployment. The relevant findings are significant to inform policies and practices on when, how and under what conditions to intervene or prevent child maltreatment. 

Income instability, which is commonly found in low-income families, is associated with worse parenting behaviors and child maltreatment [4]. Evidence showed that risk factors for child maltreatment included poor prospects of personal financial situation, short-term and long-term general economy [4] and foreclosure and local mortgage delinquency [5]. Income instability and consequent poverty was accountable for 22% of child abuse in the United Kingdom [6]. Wealth inequalities exacerbate the situation. For example, physical child abuse rate rose in states in the United States with wider income gaps during the Great Recession [7]. In Vietnam, an 8-year longitudinal study found that the higher incidence of physical child abuse among poorest households worsened further under increased socioeconomic inequalities, resulting in the poorest children having disproportionately higher risk [8].

Income instability includes job loss and unemployment, but the association between unemployment and child maltreatment was inconsistent in the global literature. A systematic review in the United Kingdom showed that reduced employment was associated with child maltreatment [9], echoed by the result of another Malaysian study [10]. In the United States, unemployment was positively associated with child maltreatment in Pennsylvania [11], but not in California [12], New York [13] and North Carolina [14]. 

In addition to the economic burden, the impact of COVID-19 on parenting practices has also been disruptive. Changes in daily routines, enhanced hygiene and infection control, school closure and remote education and suspension of social support services have increased the load on parents, increasing the risk of child maltreatment [15,16]. In addition, some studies documented the associations between income instability and parenting practices and the subsequent effect on children’s health and development [17]. However, there is no relevant research study examining the relationship between parenting difficulties and confidence on health education about COVID-19 with children and child maltreatment. 

In this study, we shed light on the mixed evidence on the association between COVID-19 and child maltreatment and attempted to address the question of whether job loss or income reduction would affect child maltreatment, including psychological aggression, corporal punishment, severe and very severe physical assaults. In addition, we examined whether parenting issues such as confidence in teaching COVID-19 and managing preventive COVID-19 behaviors with children would affect child maltreatment. To enhance the accuracy of the tested relationships between income instability or parenting issues about COVID-19 and child maltreatment, we controlled for potential confounders, including parental intimate partner violence (IPV), which was the well-known coexisting factor [18], and some COVID-19-related stressors such as parental health literacy and parent’s time staying at home during COVID-19, in the model. We hypothesized that more income instability, more difficulties and less confidence in supporting children during COVID-19 in parenting practices would be associated with more child maltreatment.

## 2. Methodology

### 2.1. Participants and Setting

We conducted a cross-sectional online survey between 29 May to 16 June 2020 with parents who were (1) residing in Hong Kong at the time of enumeration, (2) aged 18 years or above, (3) able to read Chinese, (4) married or cohabited and (5) having and living with a child or children aged under 10 years. Parents who were psychologically unprepared were excluded. 

Participant invitations were made by sending text messages to a random list of mobile phone numbers to form a panel of parents with random sampling. Prior to the start of the survey, the targeted parents were asked to read an information sheet explaining the purpose of the survey and the ethical issues. The targeted parents would then get a text message or email to access a computer-assisted online interviewing system and self-administered the questionnaire. Online informed consents were obtained before starting the survey. A pilot study was conducted with 10 persons to test and refine the questionnaire design. Of 779 recruited target parents, 600 parents completed the survey successfully, achieving a response rate of 77.0%.

### 2.2. Measurements

#### 2.2.1. Child Maltreatment during COVID-19 (Jan to April 2020)

We assessed child maltreatment, including (a) psychological aggression, (b) nonviolent disciplinary behaviors (corporal punishment), (c) severe physical assault and (d) very severe physical assault, based on Conflict Tactics Scale-Parent Child (CTSPC) scale, Chinese version [19]. We asked the parent to report any behaviors regarding his/her oldest child aged under 10 years about:

Psychological aggression: including, (i) threatened to spank or hit him/her but did not actually do it, (ii) shouted, yelled, or screamed at him/her, (iii) swore or cursed at him/her, (iv) called him/her dumb or lazy or some other name like that, (v) said you would send him/her away or kick him/her out of the house.

Corporal punishment: including, (i) spanked him/her on the bottom with your bare hand, (ii) hit him/her on the bottom with something like a belt, hairbrush, a stick or some other hard object, (iii) slapped him/her on the hand, arm, or leg, (iv) pinched him/her, (v) shook him/her.

Severe physical assault: including, (i) slapped him/her on the face or head or ears, (ii) hit him/her on some other part of the body besides the bottom with something like a belt, hairbrush, a stick or some other hard object, (iii) threw or knocked him/her down, (iv) hit him/her with a fist or kicked him/her hard, (v) slapped him/her on the face or head or ears.

Very severe physical assault: including, (i) beat him/her up, that is you hit him/her over and over as hard as you could, (ii) grabbed him/her around the neck and choked him/her, (iii) burned or scalded him/her on purpose, (iv) threatened him/her with a knife or gun, (v) beat him/her up, that is you hit him/her over and over as hard as you could.

The responses of each item were rated on a response of (i) no, (ii) yes, (iii) yes, increased, and (iv) yes, decreased. 

#### 2.2.2. Potential Confounders: COVID-19-Related Stressors

Income instability during COVID-19 (Jan to April 2020): One item asked if the parent has had any change on income. The responses were (i) job loss, (ii) large income reduction, (iii) income with half reduction, (iv) small income reduction, (v) no change or (vi) income increased.

Parenting difficulties and confidence about COVID-19 (Jan to April 2020): One item asked about the level of parenting difficulty in discussing COVID-19 information with their children. The responses were rated as 0–10, with 0 representing “no difficulty at all” and 10 representing “very difficult”. One item asked about the level of parenting confidence in implementing the preventive practice of COVID-19. The responses were rated as 0–10, with 0 representing “no confidence at all” and 10 representing “very confident”. 

Parental health literacy: The individual general health literacy was assessed by using the 16-item European Health Literacy Questionnaire short form (HLS-EU-Q16) (Chinese version) [20]. The total scores were categorized as sufficient (13–16 points), problematic (9–12) and inadequate (1–8). 

Parent’s relationship with partner during COVID-19 (Jan to April 2020): The relationship between intimate partner violence and child abuse is well-documented in the literature. We also assessed the parent’s relationship with his/her partner, in particular, for intimate partner violence (IPV). IPV was assessed by the Chinese version of the Abuse Assessment Screen [21]. It consisted of three questions to ask if the parent has been emotionally hurt, physically hurt or has been forced to have sexual activities during COVID-19 (Jan to April 2020).

Parent’s time staying at home during COVID-19 (Jan to April 2020): We wanted to know whether parent’s time staying at home was a stressor of different types of child abuse. One question was asked about how many days on average per week the parent spent most of the time staying at home (ranged from 0 to 7 days). 

Parent’s mental health distress during COVID-19: The mental health distress in the past 2 weeks was assessed by the Patient Health Questionnaire-4 (PHQ-4) [22], which provided an indicator of having anxiety and depressive symptoms.

#### 2.2.3. Sociodemographic Characteristics

Sex, age, education attainment and household income were assessed.

### 2.3. Data Analysis

Prior to data analysis, procedures of data checking were conducted in assessing the accuracy of input, missing values and checking the assumptions of regression analyses. Descriptive statistics of the frequencies, means, and standard deviation (SD) of all the study variables were performed. We performed logistic regression, in which child abuse was used as the dependent variable, and participants’ gender, age, changes of economic status during the COVID-19 epidemic, health literacy, parent’s mental health distress, parent’s IPV, level of difficulty in discussing issues related to COVID-19 with their children and level of confidence in implementing the health-related practices by their children were entered as independent variables. SPSS 24.0 was used in the data analysis. The odds ratio (OR) and 95% CI indicated the effect of each predictor and whether it met statistical significance. The final logistic regression models were evaluated by using Cox and Snell R^2^, Nagelkerke R^2^ and the Hosmer and Lemeshow test for goodness-of-fit [23].

### 2.4. Ethical Consideration

This study was approved by the Institutional Review Board of the University of Hong Kong/Hospital Authority Hong Kong, West Cluster (UW-20-347). We followed the report of STrengthening the Reporting of OBservational studies in Epidemiology (STROBE) guidelines. 

## 3. Results

The description of the participants’ characteristics is provided in Table 1. Among 600 parents, 416 participants (69.3%) were females. The participants’ mean age was 38.1 years (SD = 6.43). Half of them had monthly household income of HK$20,000 (US$2600) to HK$40,000 (US$5100), indicating that they were middle-class families. During the period of COVID-19 (Jan to April 2020), there were 151 (25.2%) participants who had their income reduced and 97 (16.1%) participants had their income reduced by half or even lost their job. The educational level of participants was generally high, with all of them having attained secondary/tertiary level or above. Majority of the participants had sufficient levels of health literacy (*n* = 494, 82.3%). There were only 61 (10%) participants having problematic levels of health literacy and 45 (7.5%) participants having inadequate levels of health literacy. 

In general, the prevalence of corporal punishment, psychological aggression, severe and very severe physical assault did not differ significantly during COVID-19 (Table 2). There were 74 parents (12.3%) that reported same or similar frequency, 14 parents (2.3%) reported increased frequency and only 1 reported decreased frequency (0.2%) in corporal punishment. For psychological aggression, 226 parents (37.7%) reported the same or similar frequency, 22 parents (3.7%) reported increased frequency and only 6 parents (1%) reported decreased frequency.

Table 3 reports the results from logistic regression analysis. Income reduction was found significantly associated with severe (OR = 3.29, 95% CI = 1.06, 10.25) and very severe physical assaults (OR = 7.69, 95% CI = 2.24, 26.41) towards children. Job loss or large income reduction was also significantly associated with severe (OR = 3.68, 95% CI = 1.33, 10.19) and very severe physical assaults (OR = 4.05, 95% CI = 1.17, 14.08) towards children. However, income reduction (OR = 0.29, 95% CI = 0.15, 0.53) and job loss (OR = 0.47, 95% CI = 0.28, 0.76) were significantly associated with less psychological aggression. Similar patterns were found in the relationship between the number of days staying at home per week and more severe (OR = 1.46, 95% CI = 1.11, 1.92), very severe physical assaults (OR = 1.47, 95% CI = 1.07, 2.03) and less psychological aggression (OR = 0.86, 95% CI = 0.76, 0.97). Income reduction or job loss was not associated with corporal punishment.

Exposure of IPV between parents was a very strong and significant factor which was associated with corporal punishment (OR = 3.72, 95% CI = 2.19, 6.34), severe (OR = 6.69, 95% CI = 2.33, 19.18) and very severe physical assaults (OR = 10.58, 95% CI = 2.85, 39.20) and psychological aggression (OR = 10.71, 95% CI = 7.04, 16.30) towards children. 

Regarding the parenting issues, having higher level of difficulty in discussing COVID-19 with children was significantly associated with more corporal punishment but no other child maltreatments. Higher level of confidence in managing preventive COVID-19 behaviors with children was found significantly associated with less child maltreatment, including corporal punishment (OR = 0.87, 95% CI = 0.76, 0.99) and very severe physical assaults (OR = 0.74, 95% CI = 0.58, 0.93). 

## 4. Discussion

This is the first study examining income reduction, job loss, parenting difficulties and health confidence during COVID-19 pandemic. Overall, there was a very mild increase in child maltreatment during the pandemic (1–3.7% increased among different types of child maltreatment) as reported from the participants. However, these initial results suggested increasing income instability and social distancing measures with longer stays at home substantially increased the risk for severe and very severe physical assault in children, even after accounting for sex and age of parents. Parents who experienced significant income reduction or job loss related to the COVID-19 pandemic were nearly two to three times as likely to physically maltreat their children during the pandemic compared with parents who had no change or even increased income. These findings were supported by another cross-sectional study conducted in the United States, with job loss amplifying the risk of child physical assaults [24]. However, in the same study, inconsistent findings were noted regarding child psychological aggression. The previous study found that parental job loss was also one of the predictors of child psychological aggression [24], while that was a protector of child psychological aggression in our present study in the Chinese population. In addition, we identified that social distancing measures made parents stay at home longer and this became a protector of child psychological aggression. With the reference of a previous systematic review [25], the possible explanation may be increased time for parent–child communication, which has prevented child psychological aggression. Therefore, the findings of the present study suggest that parent times of staying at home due to the COVID-19 pandemic has allowed for an opportunity to build positive communications with their children. However, if there are any inappropriate parenting behaviors, parent times of staying at home may also increase the risk of physical assaults.

In our study, parenting issues during the pandemic made some difference in executing corporal punishment towards children. We found that parents with higher levels of difficulty in discussing issues related to COVID-19 with children executed more corporal punishment, while parents with higher levels of confidence in implementing the health-related practices with children executed less corporal punishment. At the early stage of the COVID-19 pandemic, parenting resources on concrete tips were not available. They may not know how to explain the disease and its uncertainty and how to conduct proper infection control measures such as face mask wearing and hand hygiene to keep their children safe [16]. Our results aroused the attention of the society about protecting children from corporal punishment. Evidence showed that the reduction in reported child maltreatment allegations was largely driven by implementation of school closures during the pandemic, which in turn might put children at risk [26]. However, limited evidence demonstrated this consequence. Nevertheless, the mechanisms of parenting issues leading to corporal punishment were not examined in our study. Some studies found that parental stress towards the pandemic [27] and parental positive coping strategies [24] were the mediators leading to child maltreatment. Further study is needed to understand these mechanisms in the Chinese context. 

Of the other parental stressors, the most important risk factor remains IPV between parents. Although child maltreatment and IPV are two different forms of family violence, they are known to co-occur and overlap in risk factors between them [18,28]. Although limited studies are currently available for the COVID-19 pandemic, some evidence has shown the impact of natural disasters on family violence, especially those of lower socioeconomic status [29]. A previous study reported about the relationship between parents’ mental well-being or burnout and child maltreatment [16]. However, in our present study, inconsistent findings were identified as parents’ mental health and health literacy level were not associated with child maltreatment. Given the limited studies in the field, further studies are needed to confirm the results. 

The current study has several strengths, including the examination of child maltreatment in the early stage of the COVID-19 pandemic and investigating the risk and protective factors for different types of child maltreatment in the timely manner of data collection while parents experienced income reduction, job loss and lack of parenting resources for protecting children from the COVID-19 pandemic. Also, our study participants were recruited through a random list of mobile phone numbers to form a panel of parents in the general population in Hong Kong. However, the present study also has several limitations. Participants were asked to self-report their behaviors of child maltreatment which might under-estimate the prevalence of child maltreatment. Also, data were collected through an online platform, therefore data from those parents without smartphones or who were not familiar with online surveys might be ignored. This phenomenon was also reflected from the demographics of our participants, with generally high educational levels. Moreover, the sample size was relatively small in view of an online survey and the data collected were at one time point. Further longitudinal research with a larger sample will be necessary to verify the results and examine the trajectory of long-term implications of the pandemic on child maltreatment, parental attitudes, parental behaviors and the possible mechanism of income instability leading to child maltreatment. 

Our findings can provide evidence for some implications to future practice. In the early stage of the COVID-19 pandemic, community resources for parenting were very much limited. Training all stakeholders in community service delivery units and charity organizations about family services and child protection for infection control measures is necessary. Also, healthcare and social care providers should recognize the feelings of worry, stress and irritability in their clients who have to provide childcare, in particular, coupled with income reduction, job loss and job uncertainty. It is also recommended to pay special attention on the possibility of co-existing parental IPV and child maltreatment in the family. Online materials or mobile health technology (mHealth) can be further explored to deliver reliable parenting resources and provide social support to resolve parents’ difficulties and enhance their confidence in parenting during the pandemic.

## 5. Conclusions

Child maltreatment was found slightly increased during the COVID-19 pandemic. Income instability, such as income reduction and job loss, amplified the risk of severe and very severe physical assaults but protected children from psychological aggression. Also, confidence in teaching COVID-19 and managing preventive COVID-19 behaviors with children were significantly negatively correlated with corporal punishment during the pandemic. In addition, our study found that IPV might pose a risk for child maltreatment during the COVID-19 pandemic.

## Figures and Tables

**Table 1:** Participant characteristics.

	Total (*n* = 600)	Fathers (*n* = 184)	Mothers (*n* = 416)	*t*-Test/Mann–Whitney U Test
	*n* (%)/Mean ± SD	*n* (%)/Mean ± SD	*n* (%)/Mean ± SD	*p*-Value
**Age**	38.1 ± 6.4	40.1 ± 8.5	37.2 ± 5.0	<0.001 *
**Monthly household income**				0.34
HK$20,000 or less	40 (6.7%)	13 (7%)	27 (6.4%)	
HK$20,001–HK$40,000	302 (50.3%)	86 (46.7%)	216 (51.9%)	
HK$40,001 or above	258 (43%)	85 (46.3%)	173 (41.6%)	
**Educational attainment**				0.24
Secondary level or below	402 (67%)	117 (63.6%)	285 (68.5%)	
Tertiary level or above	198 (33%)	67 (36.4%)	131 (31.5%)	
**Economic status change during COVID-19**				0.50
No change/increased	352 (58.7%)	85 (46.2%)	267 (64.2%)	
Income reduced	151 (25.2%)	62 (33.7%)	89 (21.4%)	
Job loss or income reduced by half	97 (16.1%)	37 (20.1%)	60 (14.4%)	
**HLS-EU-Q16**				0.32
Inadequate (0–8)	45 (7.5%)	20 (10.9%)	25 (6%)	
Problematic (9–12)	61 (10.2%)	16 (8.7%)	45 (10.8%)	
Sufficient (13–16)	494 (82.3%)	148 (80.4%)	346 (83.2%)	
**Parenting**				
Level of difficulty in discussing issues related to COVID-19 with your children (0–10 level)	2.8 ± 2.1	3.0 ± 1.9	2.7 ± 2.1	0.11
Level of confidence in implementing the health-related practices by your children (0–10 level)	7.6 ± 1.9	7.1 ± 1.6	7.8 ± 1.9	<0.0001 *
**Parent time staying at home (0–7 days)**	3.5 ± 1.8	2.9 ± 1.7	3.8 ± 1.8	<0.0001 *

Note: abbreviation: SD = standard deviation; HLS-EU-Q16 = European Health Literacy Questionnaire short form; * indicates *p*-Value < 0.05.

**Table 2:** Self-reported change of child maltreatment during COVID-19 (Jan to April 2020).

	Never	Same/Similar Frequency	Increased Frequency	Decreased Frequency
	Number (%)	Number (%)	Number (%)	Number (%)
Corporal punishment	511 (85.2%)	74 (12.3%)	14 (2.3%)	1 (0.2%)
Severe physical assault	574 (95.7%)	18 (3.0%)	7 (1.2%)	1 (0.2%)
Very severe physical assault	579 (96.5%)	15 (2.5%)	6 (1.0%)	0 (0.0%)
Psychological aggression	346 (57.7%)	226 (37.7%)	22 (3.7%)	6 (1.0%)

**Table 3:** Logistic regression analysis predicting child maltreatment.

	Corporal Punishment			Severe Physical Assault			Very Severe Physical Assault			Psychological Aggression		
	OR	95% CI		OR	95% CI		OR	95% CI		OR	95%CI	
		Lower	Upper		Lower	Upper		Lower	Upper		Lower	Upper
**Sex**												
Female (Mother)	1.00			1.00			1.00			1.00		
Male (Father)	0.95	0.55	1.64	1.87	0.73	4.75	1.83	0.63	5.31	0.70	0.44	1.12
**Age**	1.02	0.98	1.05	1.03	0.97	1.08	1.05	0.99	1.11	1.05 *	1.01	1.08
**Health Literacy (HLS-EU-Q16)**	0.98	0.94	1.03	0.98	0.88	1.09	0.99	0.88	1.11	0.99	0.93	1.06
**Economic status change**												
No change/increased	1.00			1.00			1.00			1.00		
Income reduction	1.26	0.61	2.60	3.29 *	1.06	10.25	7.69 *	2.24	26.41	0.29 **	0.15	0.53
Job loss or large income reduction	1.28	0.73	2.23	3.68 *	1.33	10.19	4.05 *	1.17	14.08	0.47 *	0.28	0.76
**Number of days staying at home per week**	0.97	0.83	1.13	1.46 *	1.11	1.92	1.47 *	1.07	2.03	0.86 *	0.76	0.97
**IPV by spouse**												
No	1.00			1.00			1.00			1.00		
Yes	3.72 **	2.19	6.34	6.69 **	2.33	19.18	10.58 **	2.85	39.20	10.71 **	7.04	16.30
**Anxiety**												
No	1.00			1.00			1.00			1.00		
Yes	0.97	0.32	2.92	0.50	0.10	2.65	0.89	0.13	6.16	0.62	0.23	1.63
**Depression**												
No	1.00			1.00			1.00			1.00		
Yes	0.49	0.14	1.71	1.13	0.22	5.71	0.25	0.02	2.93	0.76	0.27	2.13
**Level of difficulty in discussing issues related to COVID-19 with your children (0–10 level)**	1.19 *	1.05	1.34	1.23	0.99	1.51	0.93	0.72	1.21	0.99	0.90	1.11
**Level of confidence in implementing the health-related practices by your children (0–10 level)**	0.87 *	0.76	0.99	0.85	0.68	1.05	0.74 *	0.58	0.93	0.90	0.80	1.02
**Goodness-of-fit statistics**												
Cox and Snell R^2^	0.10			0.07			0.08			0.31		
Nagelkerke R^2^	0.17			0.24			0.30			0.42		
Homser and Lemeshow Test	0.39			0.07			0.24			0.16		

Note: * *p* <0.05, ** *p* < 0.0001; Abbreviation: SD = standard deviation; HLS-EU-Q16 = European Health Literacy Questionnaire short form; IPV = intimate partner violence; OR = odds ratio; CI = confidence interval.

## Data Availability

The data presented in this study are available on request from the corresponding author. The data are not publicly available due to its huge size and participants’ privacy protection.
